# It's a Gas: Bioconjugation With Vapor‐Phase Reagents

**DOI:** 10.1002/chem.202503565

**Published:** 2026-02-21

**Authors:** Yuxuan Ding, Jun Ohata, Zachary T. Ball

**Affiliations:** ^1^ Department of Chemistry Rice University Houston Texas USA; ^2^ Department of Chemistry North Carolina State University Raleigh North Carolina USA

**Keywords:** bioconjugation, biphasic, gas‐phase reagents, late‐stage diversification, peptides

## Abstract

Bioconjugation is a large field with many diverse goals, needs, and challenges, that requires a broad toolbox of fundamentally different synthetic approaches. As an emerging class of bioconjugation reagents, gas molecules bring new reactivity and selectivity concepts. Beyond these fundamental questions, gas‐phase reagents may have unique advantages, such as access to porous material and structures, and diffusions/penetration differences in reaction in complex tissues or other contexts. This concept article examines vapor‐phase reagents, as well as their reactivity and selectivity, for the modification of natural peptides and proteins.

## Graphical abstract

Gaseous reagents are an emerging class of bioconjugation reagents that can offer alternative selectivity paradigms for the construction of precise bioconjugates. A variety of both inert and highly reactive gases have recently been developed for bioconjugation use. This concept article describes catalyst and reaction design concepts and the bioconjugation structures that they make possible.

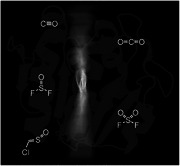

## 1 Introduction

Bioconjugation has emerged in recent years as an important area of synthetic methodology [1]. The need for selective methods for manipulating large, polyfunctional biomolecules, such as peptides and proteins, brings interesting functional‐group selectivity questions that have drawn the attention of synthetic chemists, have inspired creative methodological approaches to design or discover new selectivity paradigms in complex aqueous environments [2, 3, 4, 5, 6]. Several recent reports indicate a new emerging class of bioconjugation methods: gas‐phase reagents for bioconjugation.

There are several potential advantages of gaseous bioconjugation, depending on the specific use case. Gas‐phase reagents, by definition, are very small molecules. As such, they minimally alter the structure of a peptide/protein substrate and are probably less likely to alter biological function in unanticipated or undesired ways. Unreacted gaseous reagents may be readily separated from biomacromolecules, for example, under reduced pressure. Additionally, small, volatile reagents will typically penetrate tissues and membranes more readily than larger molecules, and so it is possible to imagine applications in living or other bulk systems. And finally, gas‐phase delivery may be an efficient way to deliver reactive reagents that would be challenging to isolate or form in situ in the aqueous environments often required for reactions of proteins.

This review focuses on recent advances involving gaseous delivery of bioconjugation reagents. We focus on cases in which the gas reagent is directly incorporated into the product and facilitates useful biopolymer functionalization, typically involving a third moiety in a multicomponent process [7]. This approach is distinct from common cellular gaseous species, such as reactive oxygen species (ROS) and NO, that react with amino acid side chains but may not provide a handle for site‐specific functionalization or elaboration.

## 2 Carbon Monoxide

Carbon monoxide (CO), primarily generated through heme degradation catalyzed by heme oxygenase [8], plays important roles in living systems, such as regulating membrane ion channel function [9] and intracellular signaling pathways [10]. While toxic at high concentrations [11], small amounts of CO are essential in living organisms. Indeed, the therapeutic use of CO has been postulated [12, 13], which led to an increasing interest in developing CO‐releasing molecules and new methods for its controlled release [14, 15]. A recent study shows that CO should have an adequate safety margin for therapeutic applications [16].

Carbon monoxide is also a versatile C‐1 building block widely used in organic synthesis, most notably for carbonylation reactions [17, 18, 19]. Despite broad use, carbon monoxide, and probably many gaseous reagents, presents several significant safety concerns in a lab setting [20]. One approach to mitigate these safety concerns is the development of two‐chamber reactors for ex situ generation of small quantities of CO for laboratory use [21]. While CO is extensively used in small molecule synthesis, the use of CO for peptide modification has been rare until quite recently.

**FIGURE 1:**
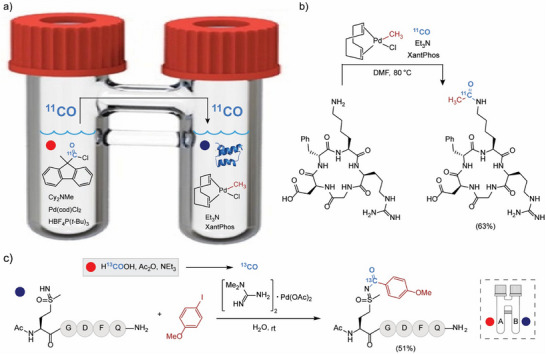
Peptide carbonylation and isotope labelling with ex situ generated CO. (a) Schematic of a two‐chamber reaction. (b) Lysine acetylation using a methyl palladium(II) complex. (c) Carbonylation of NH‐sulfoximine in polypeptides.

Acetylation of N‐terminal or lysine amines in polypeptides was achieved using a methylpalladium(II) complex in the presence of ex situ generated CO in a two‐chamber reactor (Figure 1a, b) [22]. Importantly, isotopically labeled ^11^CO precursors allow facile access to radiolabeled *N*‐^11^C‐acetylated peptides in decent yields. As carbon isotope labeling has found increasing applications in the pharmacological industry for evaluating the metabolic profiles of drug candidates [23, 24, 25], this strategy may offer new opportunities in drug development.

Sulfoximine is also demonstrated as a unique reactive handle for carbonylative cross‐coupling with aryl halides and CO [26]. The approach leverages a late‐stage one‐step oxidation of methionine residues to afford NH‐sulfoximines in polypeptides, followed by a Pd‐catalyzed carbonylation of methionine sulfoximine‐containing peptides with an aryl iodide in water (Figure 1c) [27]. As above, this reaction also utilized a two‐chamber reactor for ex situ CO generation, and the use of an isotopically labeled ^13^CO source allows simultaneous peptide functionalization and isotope labeling.

**FIGURE 2:**
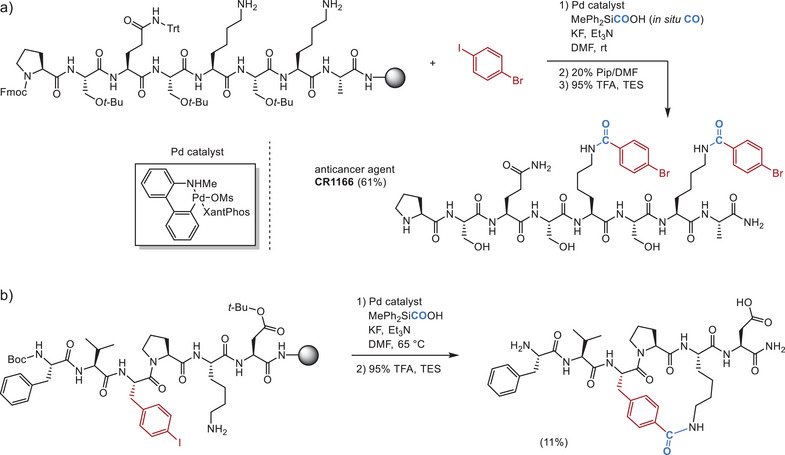
Aminocarbonylation with in situ CO for peptide modification and cyclization. (a) Lysine carbonylation with aryl iodides in an anticancer peptide. (b) Intramolecular carbonylation for peptide cyclization.

Aminocarbonylation has also been used for on‐resin functionalization with a palladacycle precatalyst (Figure 2) [28]. The use of a silacarboxylic acid as an in situ CO precursor allows the reaction to be set up in a single vial. The incorporation of a noncanonical amino acid, 4‐iodophenylalanine, allows carbonylative cyclization, providing access to a cyclic peptide in moderate yield (Figure 2b). The method shows good tolerance of several (hetero)aryl iodides and a variety of amino acids protected with common protecting groups for solid‐phase peptide synthesis (SPPS).

## 3 Carbon Dioxide

Carbon dioxide (CO_2_) is typically considered an inert molecule with limited synthetic applications beyond reaction with carbanions. However, it has been shown that the electrophilicity of CO_2_ can be similar to aryl isocyanate derivatives [29]. In living systems, CO_2_ has a variety of roles beyond pH‐regulation via carbonic acid [30, 31], and growing evidence suggests amine carboxylation with CO_2_ to reversibly form carbamic acid groups on lysine residues constitutes an important but poorly understood post‐translational modification [32, 33, 34]. Perhaps with this in mind, CO_2_ has recently been used for chemical modifications of peptides and proteins as well.

**FIGURE 3:**
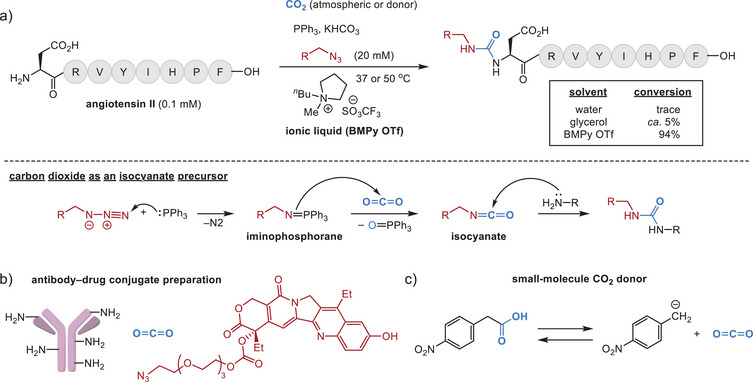
Carbon dioxide as a reactant for the urea‐forming amine‐azide coupling reaction. (a) Reaction scheme of a model peptide angiotensin II. The reaction conversions in different media are based on the data in the original report [43]. A brief reaction mechanism is shown below the dotted line. BMPy OTf: 1,1‐butylmethylpyrrolidinium trifluoromethanesulfonate. (b) Application of the CO_2_‐based bioconjugation for antibody–drug conjugate preparation. Azide‐containing small molecule drug (SN‐38) is shown on the right. (c) Arylacetic acid derivatives as small molecule CO_2_ donors for the amine azide coupling reaction.

Using ionic liquids as a protein‐compatible bioconjugation media allows novel CO_2_ reactivity patterns. As one of the ways to make use of CO_2_ as a building block, iminophosphorane or aza‐Wittig reagents facilitate urea formation with small molecules [35, 36, 37]. However, aprotic, anhydrous solvents are typically required to suppress the Staudinger reduction pathway in water, and successful use of an iminophosphorane intermediate in aqueous solutions is limited to intramolecular processes (e.g., CO_2_ detection [38] and Staudinger ligation [39] in cells). Ionic liquids—a class of salts with melting points of <100°C—have unique properties as a highly polar yet aprotic media, and these unique properties allow effective labeling of N‐terminal and lysine amines via isocyanate intermediates from CO_2_ and an iminophosphorane generated from an alkyl azide (Figure 3) [40]. This approach proved feasible because of high solubility of both proteins and CO_2_ in ionic liquids [41]. The chemoselectivity in favor of alkylamine groups was ascribed to the inherent reactivity preference of the isocyanate formed in situ [42], and this attribute has been leveraged for chemical modification of various substrates including a monoclonal antibody [43, 44], DNA aptamer [45, 46], polysaccharide [47], and cell lysate [47]. At small scales (e.g., low µM concentration of peptides), the ionic liquid‐based reactions proceed efficiently with an atmospheric level of CO_2_, and the same authors developed a convenient way to perform the bioconjugation reaction at a larger scale by employing a small‐molecule CO_2_ donor as well [48].

## 4 Sulfur Fluorides

Sulfur fluorides, such as sulfuryl fluoride (SO_2_F_2_) and thionyl fluoride (SOF_2_), are useful reagent in organic synthesis [49, 50]. The development of sulfur (VI) fluoride exchange (SuFEx) click chemistry [51] further expanded their utility to include chemical biology and medicinal chemistry [52, 53, 54]. Despite unique reactivity, the gaseous and toxic nature of SO_2_F_2_ and SOF_2_ have impeded broad adoption. With the development of specialized equipment for gas‐phase reactions and new precursors for SO_2_F_2_ and SOF_2_ generation, the applications of these reagents in peptide modification are explored in recent years.

**FIGURE 4:**
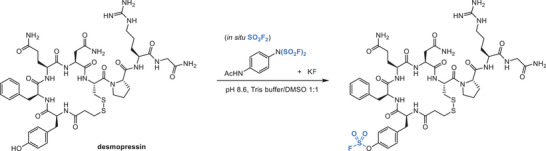
Tyrosine modification with SO_2_F_2_ generated in situ from AISF and KF.

For example, (4‐(acetylamino)phenyl)imidodisulfuryl difluoride (**AISF**) is a bench‐stable, crystalline reagent that offers an alternative to SO_2_F_2_ [55], allowing synthesis of fluorosulfates from alcohols or amines. Interestingly, **AISF** generates SO_2_F_2_ gas in situ in the presence of potassium fluoride, achieving tyrosine modification in a bioactive peptide and giving the fluorosulfate product (Figure 4) [56]. The in situ generation of SO_2_F_2_ as the reactive species was confirmed by ^19^F NMR.

**FIGURE 5:**
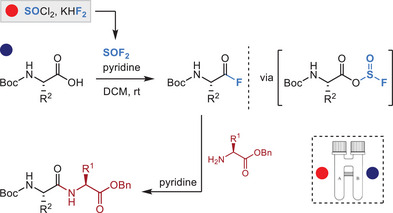
Peptide synthesis with ex situ generated SOF_2_.

Relative to SO_2_F_2_, SOF_2_ is more reactive and can be used for efficient carboxylate activation. A novel protocol was developed to produce SOF_2_ ex situ from SOCl_2_ and a fluoride salt for carboxylic acid activation in a two‐chamber reactor. The resulting sulfite intermediates were readily converted into acyl fluorides, which could then react with amines to form amide bonds. Amino acid coupling under these conditions produced a range of dipeptide products with minimal to no epimerization (Figure 5).

It is noteworthy that this method is also amenable to liquid‐ and solid‐phase peptide synthesis to give polypeptides up to 10‐mers in decent yields without the need for column chromatography (for liquid phase synthesis) [57].

## 5 Chlorosulfine

**FIGURE 6:**
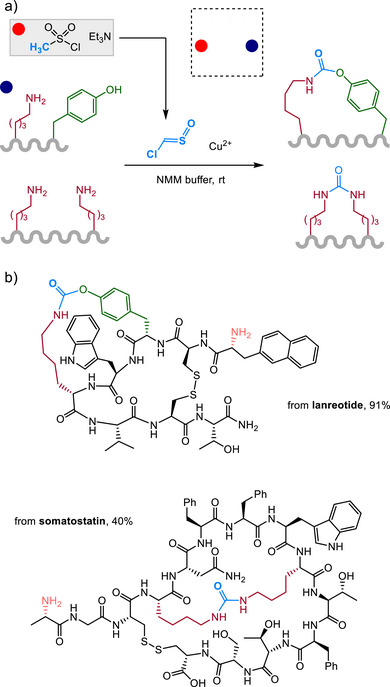
(a) Chlorosulfine generation for peptide macrocyclization. (b) Polycyclic analogues of bioactive peptides accessed by selective macrocyclization of unprotected peptides.

The use of ex situ gas generation in two‐chambered vessels has also facilitated the discovery and use of other reactive sulfur species with unique bioconjugation capabilities. A chloride‐substituted analogue of the simplest C_1_ sulfine (also called a thiocarbonyl S‐oxide) has recently been implicated as the key gaseous intermediate in a linchpin, multicomponent coupling of peptides and proteins, in which urea or carbamate products are formed from linking two different N‐ or O‐nucleophiles [58, 59]. Treatment of unprotected peptides in aqueous buffer with an ex situ source of chlorosulfine (Cl(H)C═S═O) in the presence of a copper(II) salt efficiently produces macrocyclic products (Figure 6a) [58]. Cyclization to a urea or carbamate incorporates a carbon atom from the chlorosulfine, and links amino side chains (typically lysine) with amino, phenol, or aniline moieties. Several examples of macrocyclic peptide natural products were studies as substrates, affording complex polycyclic analogues.

A salient feature of this reactivity is the surprising functional‐group selectivity observed. Cyclization at N‐terminal amines is strongly disfavored, relative to lysine side‐chain amine groups, and even tyrosine phenol groups were shown to participate preferentially in the presence of N‐terminal amine groups. This selectivity is opposite that typically observed in many amine‐selective bioconjugations, in which N‐terminal selectivity is often observed on the basis of pKa differences. Opposite selectivity in this case may be indicative of unique mechanistic pathways, or of the role of the copper mediator. While functional group specificity in complex sequences may be difficult to predict a priori, these examples indicate that chlorosulfine does tend to give selective reactivity and can deliver direct formation of cyclized analogues of bioactive peptides with unique linkage architecture not available with other reagents (Figure 6b) [58, 60, 61].

**FIGURE 7:**
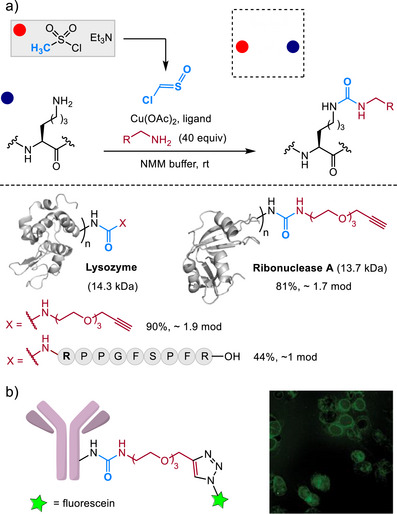
(a) Intermolecular bioconjugation with chlorosulfine and amine reagents. (b) Cell imaging with a fluorophore‐modified antibody produced by chlorosulfine labeling. Adapted in part with permission [59]. Copyright 2024 American Chemical Society.

The initial study was extended to intermolecular bioconjugation of peptides and proteins [59]. Modifying lysine side chains with chlorosulfine in the presence of other amine reagents (Figure 7a) allowed the construction of proteins labeled with biorthogonal handles, bioactive peptides, or fluorophores for antibody imaging applications (Figure 7b).

In addition to the recent development of the sulfuryl fluoride‐ and chlorosulfine‐based bioconjugation approaches, other sulfur‐based gases have potential for use in chemical modification of proteins. Hydrogen sulfide (H_2_S) is a gasotransmitter with diverse reactivity, and the intermediacy of a hydropersulfide group (RSSH) on a cysteine residue is postulated in various biological events [62]. Indeed, in vitro modification of cysteine residues to hydropersulfide can be achieved by using a simple sulfide salt (Na_2_S) [63]. Methanethiol (H_3_CSH) is known to form disulfide bonds on a cysteine residues, but has only been studied in the context of redox biology [64]. The main research focus of sulfur dioxide (SO_2_) has been its use as a food preservative known to cause protein aggregation through its reaction with disulfide bonds [65]. However, SO_2_‐based reactions in synthetic organic chemistry can be facilitated by its solid‐form surrogate (DABSO) [66], and this new capability may be an indication of promising potential for biomolecule functionalization [67]. Lastly, trifluoromethanesulfonyl fluoride (F_3_CSO_2_F) is a sulfur‐based gas with growing use in synthetic organic chemistry including SuFEx reactions [68], and so extension of this gas to SuFEx‐type bioconjugation may be possible [54].

## 6 Formaldehyde

**FIGURE 8:**
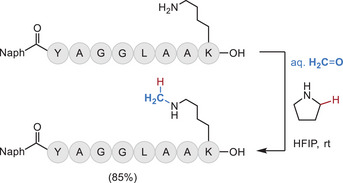
Lysine monomethylation with formaldehyde/pyrrolidine.

Formaldehyde is a gas at room temperature and has a long history as a protein crosslinking agent. Decades ago, formaldehyde radiolabeled with ^11^C was synthesized and delivered in the gas phase to aqueous protein solutions for radiolabeling purposes [69]. Gaseous applications of formaldehyde are rare, however. More commonly, solid paraformaldehyde dissolved in water is sufficient for bioconjugation applications, such as Mannich reactions at tyrosine [70] and condensation reactions of arginine and lysine [61, 71]. Quite recently, it was reported that formaldehyde could facilitate selective lysine monomethylation, via a formal reductive amination process, without a traditional hydride source (Figure 8) [72]. The mechanisms of formaldehyde reactivity with amino acids has recently been reviewed [73].

## 7 Other Gases

Several other gases that react with proteins and peptides, in some cases provide biologically interesting, relevant reactivity and product structures, which nonetheless have not been used for functionalizing biomolecules with useful handles or labels. For example, acetaldehyde (H_3_CCHO) has a high vapor pressure, and its in vivo formation from ethanol and covalent attachment to proteins is well known [74, 75]. Yet acetaldehyde's use in bioconjugation use is virtually nonexistent. Phosgene (COCl_2_) gas can be used in peptide synthesis [76, 77], although its solid precursor, triphosgene, and other alternatives such as carbonyldiimidazole (CDI) are more widely employed due to the notorious toxicity of phosgene itself. Because of its usefulness for radiolabeling, the use of hydrogen cyanide (HCN) for peptide modification is primarily centered around isotopic radiolabeling with ^11^C‐labeled HCN [78]. The simple fluorine‐containing gasses F_2_ and HF have similarly been used for ^18^F radiolabeling [79]. Recently, palladium cross coupling with labeled HCN has been used to label unprotected peptides [80]. While isocyanate and its derivatives are common amine‐labeling reagents, protein carbamoylation by the parent isocyanic acid (HNCO) has been studied only as a gaseous environmental pollutant [81, 82]. In a similar vein, diazo derivatives have been widely used for chemoselective bioconjugation strategies (e.g., aspartic acid [83], lysine [84], cysteine [85], and tryptophan [86]). Some small diazo compounds are gases in pure form, such as diazomethane (H_2_CN_2_) and difluorodiazoethane (HF_2_CHN_2_) [87] but these compounds are typically employed as solutions in peptide chemistry [88] and have not been used for bioconjugation purposes to date. It is interesting to note that some hydrocarbons, such as ethylene, propylene, or acetylene have not been used in bioconjugation, yet such hydrocarbons are known to play important roles in cellular environments [89] and could conceivably be used in metal‐catalyzed processes in water [90].

A second broad category includes reactive nitrogen and oxygen species (RNS and ROS, respectively), which react with biopolymers, but typically do not facilitate labeling with moieties or structures of interest. As quintessential gasotransmitters, nitric oxide (NO) and peroxynitrite (ONOO) are able to cause post‐translational modifications (PTMs) of proteins on cysteine (*S*‐nitrosylation) [91] and tyrosine (nitration) [92], respectively. Possibly, their extremely high reactivities might have prevented its wide applications for bioconjugation to date. It is plausible, perhaps, to imagine two‐step bioconjugation strategies targeting nitrosyl compounds or other products of RNS reactivity. Additionally, hydrazoic acid (HN_3_) has been used in azide‐alkyne cycloaddition on peptides [93], though reports suggest caution in employing HN_3_ due to stability and toxicity concerns [94, 95]. In contrast, dinitrogen (N_2_) and ammonia (NH_3_) are probably too inert for bioconjugation applications, as the main use of those gaseous molecules by natural systems is not for covalent bond formation on canonical amino acids on peptides and proteins but other types of processes [96, 97]. It should be noted that aqueous ammonia solution was used for traceless release of a biotin group from protein conjugates in a cellular sample, although the role of ammonia in that case may be simply as a volatile base to raise solution pH [98].

Oxidative reactions are increasingly employed for bioconjugation processes, and ROS and other small, gaseous oxidizers have contributed to the growth of these methods. Various chemical reactions of ROS with proteins are known in natural systems [99], and photocatalytic bioconjugation by superoxide radical (O_2_^•–^) has been employed in useful and well defined bioconjugation reactions [100]. Recent review articles provide a good treatment of superoxide radical‐mediated bioconjugation [101, 102]. ROS have also been studied in oxidative cleavage of protein backbones [103]. The physical and chemical properties of most ROS, including O_2_^•–^ and hydroxyl radical (^•^OH), are not fully understood due to their transient nature, and their behaviors as an isolated gas remain elusive. Oxidative reactions on proteins can be also caused by dichlorine (Cl_2_) and hypochlorous acid (HClO) [104]. The use of such chlorine‐based oxidizing gases, however, has not been common in bioconjugation strategies.

## 8 Summary and Outlook

Appreciation for the biological importance of gasotransmitters dates back decades, accelerating in the years since the landmark 1998 Nobel Prize for nitric oxide cell signaling [105]. This review demonstrates that gaseous reagents have made new impacts on polypeptide bioconjugation development in recent years. Bioconjugation research has devised ingenious ways to implement selective, efficient reactions of polypeptides with gaseous molecules including unique reaction setup, use of small molecule donors, nonaqueous solvents, and catalytic strategies as described in this article. The literature review also highlights recent trends, potential future directions, and remaining challenges of these bioconjugation approaches. It seems certain that other gas molecules, such as silicon‐ and phosphorous‐based structures, remain unexplored and could provide exciting opportunities for unique selectivity. Future work may well focus on addressing practical challenges, including control of gas stoichiometry and scalability questions related to 2‐phase reactions. In addition, reproducibility or safety concerns have not yet been fully elucidated. Gas‐phase reagents present unique safety concerns. In most of the methods highlighted in this review, only small quantities of gaseous reagents are generated either ex situ or in situ, thereby avoiding the use of pressurized gas cylinders. However, scale up of these methods would likely present additional challenges and safety concerns, and few examples discussing those scale‐up issues have been reported. Notably, gas‐based reactions are growing strategies in synthetic organic chemistry as well [106], and, as such, reactivity discoveries in one field will undoubtedly have useful application in the other.

## Conflicts of Interest

The authors declare no conflicts of interest.

## Data Availability

Data sharing not applicable—no new data generated
